# Supplementary material for: Causal interplay between lactose intolerance and gut microbiota: a combined bidirectional Mendelian randomization and in vivo validation study
Source: Front Nutr. 2026 Jun 1;13:1803337. doi: 10.3389/fnut.2026.1803337 (PMC13265576; doi:10.3389/fnut.2026.1803337)
Supplement: Supplementary file 1 [file Data_Sheet_1.zip › supplementary materials/Forward/leave one out cross validation/ebi-a-GCST90027501.finngen_R12_E4_LACTONAS.pdf]

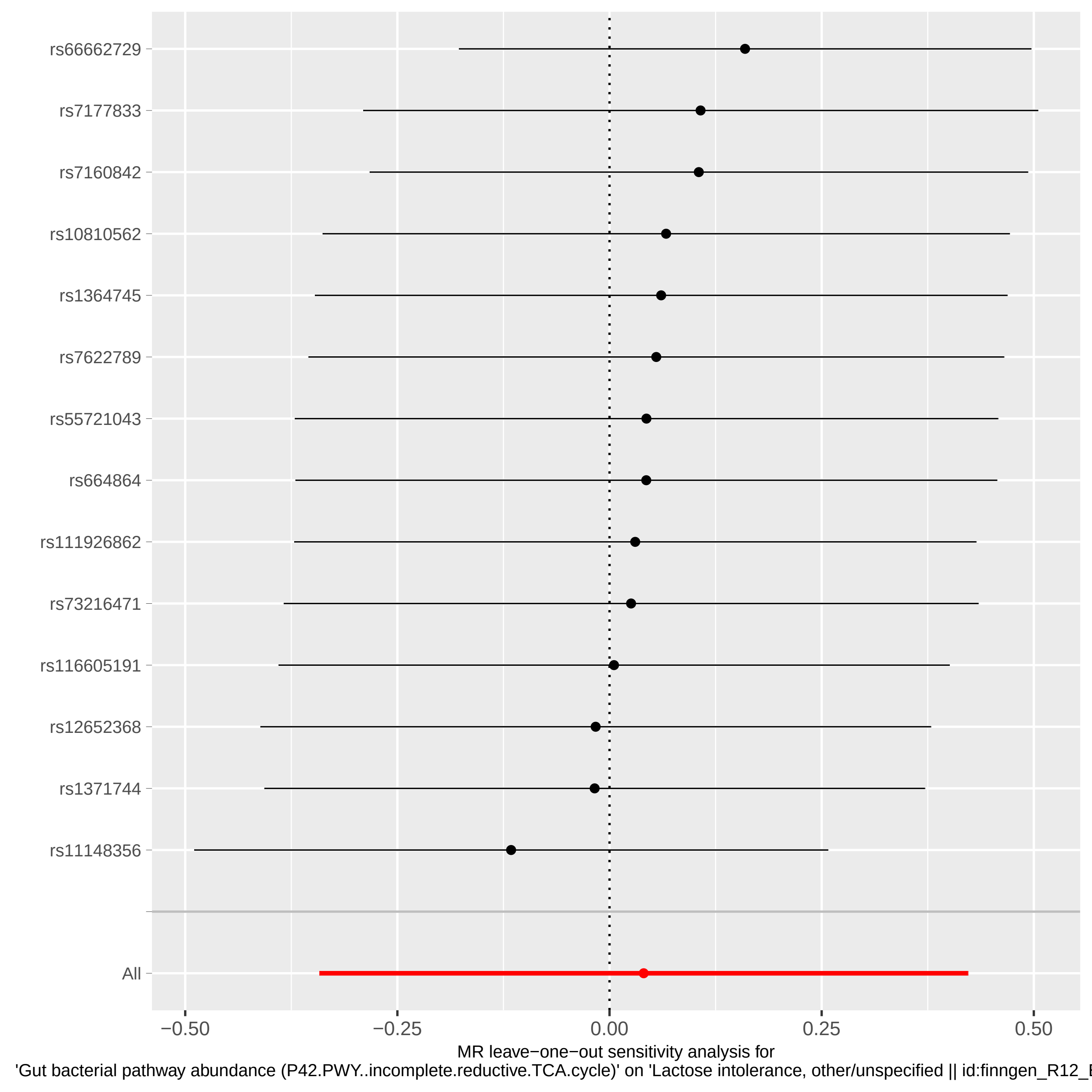

rs66662729
rs7177833
rs7160842
rs10810562
rs1364745
rs7622789
rs55721043
rs664864
rs111926862
rs73216471
rs116605191
rs12652368
rs1371744
rs11148356
All
−0.50
−0.25
0.00
0.25
0.50
MR leave−one−out sensitivity analysis for
'Gut bacterial pathway abundance (P42.PWY..incomplete.reductive.TCA.cycle)' on 'Lactose intolerance, other/unspecified || id:finngen_R12_
